# Anesthesia management of patients undergoing hyperthermic isolated limb perfusion with melphalan for melanoma treatment: an analysis of 17 cases

**DOI:** 10.1186/1471-2253-13-15

**Published:** 2013-07-17

**Authors:** Heiner Ruschulte, Serena Shi, William W Tseng, Kerstin Kolodzie, Philip C Crawford, Darren B Schneider, Mohammed Kashani-Sabet, David Minor, Christian Apfel, Stanley PL Leong

**Affiliations:** 1 Department of Anesthesia & Perioperative Care, California Pacific Medical Center, San Francisco, CA, USA; 2 Department of Surgery, California Pacific Medical Center, San Francisco, CA, USA; 3 UCSF Adult Perfusion Service, California Pacific Medical Center, San Francisco, CA, USA; 4 California Pacific Medical Center (CPMC), San Francisco, CA, USA; 5 Department of Anesthesiology & Intensive Care Medicine, Hannover Medical School Medical Center, Hannover, Germany; 6 Department of Surgical Oncology, University of Texas, M.D. Anderson Cancer Center, Houston, TX, USA; 7 Division of Vascular and Endovascular Surgery, Weil Cornell Medical College, New York, NY, USA; 8 Center for Melanoma Research and Treatment, California Pacific Medical Center, California Pacific Medical Center Research Institute, 2340 Clay Street, 2nd Floor, San Francisco, CA 94115, USA

**Keywords:** In-transit metastatic melanoma, Isolated heated limb perfusion, Melphalan, Anesthesia, Invasive monitoring

## Abstract

**Background:**

Hyperthermic isolated limb perfusion (HILP) is used for patients with intractable or extensive in-transit metastatic melanoma of the limb to deliver high concentrations of cytotoxic agents to the affected limb and offers a treatment option in a disease stage with a poor prognosis when no treatment is given.

**Methods:**

In a retrospective chart review of 17 cases, we studied the anesthetic and hemodynamic changes during HILP and its management.

**Results:**

HILP was well tolerated except in one case that is described herein. We present summary data of all cases undergoing upper and lower limb perfusion, discuss our current clinical practice of preoperative, perioperative and intraoperative patient care including the management of HILP circuit.

**Conclusion:**

HILP is a challenging procedure, and requires a team effort including the surgical team, anesthesia care providers, perfusionists and nurses. Intraoperatively, invasive hemodynamic and metabolic monitoring is indispensable to manage significant hemodynamic and metabolic changes due to fluid shifts and release of cytokines.

## Background

HILP is a cancer treatment for patients with unresectable recurrent in-transit metastases of cutaneous melanoma of the limb [1-3]. The technique delivers a high concentration of cytotoxic agents regionally to the affected limb and, thus, minimizes systemic toxicity [2-4]. Since its first description in 1950s [5], the method has gained acceptance as an established treatment modality with high reported complete response rates, ranging from 40-85%, after HILP treatment [4-8].

HILP is technically complex and demands close collaboration amongst surgeons, anesthesiologists, perfusionists and nurses. The procedure involves the use of an extracorporeal circuit, incorporating a blood pump and an oxygenator, to heat the perfusate and increase the oxygen tensions before delivery to the isolated limb [1,4,9]. During the HILP procedure, a dynamic fluid shift exists between the systemic vascular compartment and the vascular compartment of the isolated limb, especially when the patient is placed upon and disconnected from the extracorporeal circuit [2,4]. Potential life-threatening complications, such as acute hypovolemia after HILP, have been reported [8,10]. However, anesthetic management of the procedure has not been much discussed, and no standard of practice has been established [11,12].

To gain a better understanding of hemodynamic changes during the HILP procedure, we analyzed the anesthetic experience during the HILP procedures at Moffitt Long Hospital, University of California San Francisco (UCSF), CA, from 7/1/2006 to 7/1/2009. The information collected was used to establish a perioperative hemodynamic profile so that an intraoperative protocol during HILP could be established.

## Methods

### Patients

The study was approved by the Committee on Human Research (CHR) at UCSF. We performed a retrospective chart review study to establish the intraoperative hemodynamic profiles of patients during surgery and HILP.

All consecutive patients with unresectable metastatic melanoma to isolated limb undergoing HILP treatment between 7/1/2006 to 7/1/2009 at UCSF were included. We examined retrospectively the perioperative anesthetic data and reviewed the intraoperative hemodynamic changes in association with major stages of the HILP procedure, fluid replacement, and medication administrated.

### Anesthesia care and monitoring

All patients were seen for preoperative evaluation at the UCSF Melanoma Clinic including Dermatology, Surgical Oncology and Medical Oncology. The patients were also seen by a vascular surgeon, who participated in the cannulation of the vessels during the perfusion procedure. For preoperative anesthesiological evaluation, they were seen at the hospital’s Prepare Clinic where history and physical were taken. Blood was drawn for CBC, electrolytes and liver function studies. Also, ECG and chest x-ray were ordered. The anesthetic regimen was explained, anesthesia-relevant instruction given and consent obtained.

On the day of surgery, anesthesia was induced after premedication with 1–2 mg of midazolam under standard monitoring. Induction agents were propofol 1–2 mg/kg body weight (= BW), fentanyl 0.5 - 1 mcg/kg BW and rocuronium 0.6 mg/kg BW for neuromuscular blockade. After orotracheal intubation, an arterial line, a central venous line, a nasogastric tube and a urinary catheter were inserted. In addition to the relatively small (20G or 18G) peripheral intravenous cannula used for induction, one or two more large-bore intravenous cannulas (14G or 16G) were placed as access for volume replacement. Between three and five units of packed red blood cells (RBC) were ready for each patient.

Anesthesia was maintained with either desflurane or sevoflurane using low flow (Aisys, Datex Ohmeda, Waukesha, WI, USA) with an FiO_2_ between 0.5 to 1.0. Fentanyl was given in increments of 0.5 to 2 mcg/kg BW per hour. If necessary for surgical nerve monitoring, further administration of neuromuscular blockers was avoided. Phenylephrine was occasionally administered in the isolated limb or in the systemic circulation to regulate pressure differentials and prevent communication between the separate “circuits”. Otherwise, standard medications including pressors were at hand in the anesthesia cart.

All monitoring parameters and anesthesia machine data were recorded electronically (Picis, Wakefield, MA; USA). All anesthetics were carried out by or under the continuous supervision of experienced and qualified anesthesiologists.

At the end of the procedure the administration of inhaled anesthetic was stopped, and after reversal of neuromuscular blockade, and otherwise fulfilling the criteria for safe extubation, the trachea was extubated in each patient. The patients were then transferred to an intensive care unit (ICU) for overnight monitoring.

### Surgical treatment

After induction of anesthesia, the operative site (axilla or groin) and the entire ipsilateral limb was prepared and draped in a sterile fashion. A radical lymph node dissection was carried out, if not previously performed. For the upper extremity, this comprised of complete removal of lymph nodes in levels I-III. For the lower extremity, superficial groin dissection up to the inguinal ligament was performed in all patients; depending on preoperative imaging results, in selected patients, deep/pelvic dissection through a retroperitoneal exposure was also performed. After lymph node dissection, the axillary artery and vein (for upper extremity) or femoral artery and vein (for lower extremity) were exposed and side branches either ligated or clamped prior to cannulation. The sizes of the catheters were adjusted according to the sizes of the vessels. Systemic heparin (300 i.u./kg BW) was given and the vessels were cannulated. The target ACT was 480 seconds (ACT Plus, Medtronic, Inc., Minneapolis, MN). Heparin was administered as needed to maintain this value throughout the perfusion. Steinman pins were placed in the subcutaneous tissue to secure the Esmarch tourniquet around the limb, proximal to the cannulation site.

Target limb-site temperatures were 40°C, with perfusate temperatures maintained between 40-42°C to achieve these temperatures. Tumor temperature was maintained at 40°C minimum. Extremity temperature targets were reached within 40 to 70 minutes in all cases. Upon achieving target limb and tumor temperatures, a calculated Melphalan dosage was administered intra-arterially, directly via the circuit reservoir. For upper extremity 13mg Melphalan/liter limb volume (by water replacement method) were calculated, for lower extremity 10 mg/ liter limb volume were calculated, respectively, the maximum dose being limited to 120 mg even if the calculated volume was higher. Hyperthermic perfusion was then maintained for 60 minutes.

Arterial blood gases (ABG) were monitored throughout HILP: Limb and systemic samples were taken alternately. ACT values were determined every 30 minutes in both limb and systemic circulations to prevent thrombus formation. A high extremity PaO_2_ was maintained (400–550 mmHg) with attempted maintenance of normal PaCO_2_.

Washout of the treated limb was begun after 60 minutes of HILP after injection of Melphalan at 40°C of the limb. Approximately 1,000 – 3,000 ml was removed from the limb by diverting flow from the venous reservoir of the circuit into waste canisters. This volume was replaced with an equal amount of crystalloid solution, followed by 500–1,000 ml colloidal volume replacement solution (starch or gelatine-based) and packed red blood cells, if indicated by clinical presentation and bedside lab testing. An attempt was made to visualize a noticeable drop in the hemoglobin level of the discarded perfusate with a target of a "near-clear" effluent. Tourniquet occlusion was released upon completion of the washout.

At the conclusion of limb perfusion, the artery and vein were decannulated and the arteriotomy and venotomy sites, respectively, were closed using running polypropylene (Prolene) sutures. Protamine was given as a test dose (0.2 – 0.5 mg), and then administered (0.5 - 1 mg/100 units of Heparin) to fully reverse prior heparinization. Muscle (i.e. sartorius, for lower extremity) flaps were rotated to provide additional soft tissue coverage of the exposed vessels. The operative site was irrigated, hemostasis was achieved, and catheter drains (Jackson-Pratt) were left in place. The overlying fascia and subcutaneous tissues were closed using a running, absorbable polyglycolic acid suture. The skin was closed using staples or sutures and local anesthetic (0.25% bupivacaine, 10 – 15 mL) was injected subcutaneously.

### Limb perfusion circuit

An integrated infant membrane oxygenator (Capiox Baby Rx05, Terumo Cardiovascular Systems Corp., Ann Arbor, MI) was used for its low volume prime requirement and high efficiency heat exchange capability. A specially modified tubing pack (1K35R, Medtronic, Inc., Minneapolis, MN) with a ¼” tubing circuit was standard. No arterial filter was used. Arterial line monitoring was used to prevent inadvertent pressurization. A “Y” connector with attached tubing was placed in the venous line to allow for limb exsanguination during the washout phase. A standard roller pump adjusted to occlusive (HLM w/Perfusion Controller, Cobe Laboratories, Inc., Lakewood, CO) was used as the main perfusion console, and a modified heater-cooler adjusted to exceed 42°C (Hemotherm 400 M, Cincinnati Sub Zero Products, Inc. Cincinnati, OH) was used as the warming source.

Because of the low flow requirements of HILP, all cannulation was achieved by the discretionary use of venous/vena-caval type cannulae. Venous cannulae are longer than many arterial styles, and allow for deeper insertion in arterial vessels, providing better arterial isolation. Lower extremity cannulation of femoral/iliac arteries was accomplished with 12-16Fr. Cannulae (DLP 66112–66116, Medtronic, Inc., Minneapolis, MN). Lower extremity cannulation of femoral/iliac veins was accomplished with 14-20Fr. cannulae (DLP 66114–66120, Medtronic, Inc., Minneapolis, MN,). Upper extremity cannulation of brachial/cephalic arteries was accomplished with 10-14Fr. cannulae (DLP 66110–66114, Medtronic, Inc., Minneapolis, MN,). Upper extremity venous cannulation was accomplished with 12-16Fr. cannulae (DLP 66112–66116, Medtronic, Inc., Minneapolis, MN,).

Arterial and venous blood temperatures in the extremity sites, esophagus, tumor, and warming source (water) were monitored. The perfusion circuit was primed with 400 ml of a balanced electrolyte solution (PlasmaLyte-A, Baxter Healthcare, Inc., Deerfield, IL), and the perfusate was pre-warmed to 43 C. Blood flow ranges were calculated based upon known values for normal extremity distribution: arm at 9% of cardiac output (CO), and leg at 18% CO. Flows for all procedures reported ranged between 300 and 1,100 ml/min (average 386 ml/min at upper extremity, average 880 ml/min at lower extremity) with higher flow rates in the femoral/iliac circuit.

### Design and data collection

Anesthetic and hospital records were used to collect demographic data and general medical background information of each subject, including age, sex, body mass index, American Society of Anesthesiologists (ASA) classification, melanoma stages, associated medical diseases, surgical and limb perfusion technique, perioperative morbidities, and 3 month status.

The anesthetic and perfusion records were used to obtain data such as anesthetic technique, monitoring, anesthetic medication administration, intraoperative fluid and transfusion management, and perioperative laboratory values.

Hemodynamic indices used for the study included heart rate (HR), systolic blood pressure (SBP), diastolic blood pressure (DBP), mean arterial pressure (MAP), central venous pressure (CVP), urine output, temperature, estimated blood loss, blood product transfused, and total fluid replacement, For statistical analysis, we calculated shock index values using the HR values divided by SBP values from the records.

Hemodynamic data were collected and analyzed in relationship to major stages of the HILP procedure, like beginning of the surgery, clamping of infusion vessels, initiation of extracorporeal circuit, melphalan infusion, discontinuation of extracorporal circuit, exsanguination of the operated limb and end of surgery.

A waiver of consent was requested with the CHR application. All data were obtained in the form of a chart review using established medical and electronic records available at UCSF. No additional data were created as a result of the study. All data collected would have been already recorded by the clinicians in line with standard clinical practice at the time of the HILP treatment. Usual perioperative medical care was unaffected by study participation. No further patient contact was needed as a part of the study. No identifiers were included in the data collection process or included in research records. Information was only available to research group members directly involved in this study. The 3 month follow-up data were collected from the charts or electronic records. All data was kept in a secure, password-protected environment

### Data analysis

Data acquired were analyzed using Excel and statistical software STATA. Data were summarized as mean (±SD) and analyzed using Spearman’s rank correlation test where appropriate. Hemodynamic profiles were created by correlating the mean values (±SD) of various hemodynamic indices in relationship to major stages of the HILP procedure. A p-value of < 0.05 was considered significant.

## Results

From 2006 to 2009, seventeen patients with Stage III or in-transit melanoma of the limb received HILP (see Table 1). All patients were ASA classification II or III. Age ranked between 42 and 84 years (average 66.06 years), 8 patients were female, 9 were male. Twelve patients were treated for lower extremity disease and five patients for upper extremity disease. Two patients had melanoma of an unknown primary site.

**Table 1:** Patient demographics, site of procedure

	**Initital presentation**			**Isolated limb perfusion and follow-up**				
**Pt. no.**	**Primary site **	**Breaslow thickness (mm)**	**Staging**	**Perfusion site**	**Intraoperative complications**	**Acute limb toxicity grade**	**Other postop.complications (< 30 days)**	**Diseasefree at 6 months**
**1**	Unknown	Unknown	III C	R LE	-	II	-	Y
**2**	L UE,	2.0	III C	L UE	-	II	Wound dehiscence	Y
**3**	L UE, forearm	2.9	III B	L UE	-	II	Atrial fibrillation	N
**4**	L LE	3.8	III C / IV	L LE	Hypotension	I	-	Y
**5**	L LE	3.1	III	L LE	-	I	Wound infection	N
**6**	L LE, foot	unknown	III	L LE	Hypotension, arrest	III	Wound infection, dehiscence	N
**7**	L LE, calf	Unknown	III C	L LE	Hypotension	II	-	N
**8**	L LE, thigh	4.1	III C	L LE	-	II	-	Y
**9**	Unknown	Unknown	III	R LE	-	II	-	Y
**10**	L UE	4.3	III B	L UE	-	II	Wound seroma	N
**11**	L LE, thigh	4.4	III C	L LE	-	II	-	Y
**12**	L LE, calf	1.5	III C	L LE	-	II	-	Y
**13**	L UE,thumb	0.5 (MIS)	III C	L UE	-	II	-	Y
**14**	L LE	unknown	III C	L LE	-	II	Ileus	N
**15**	R LE, ankle	1.5	III	R LE	-	II	-	Y
**16**	L LE, ankle	5.5	III	L LE	-	II	-	N
**17**	R UE	1.1	III C	R UE	-	I	-	N

(*LE* lower extremitiy, *UE*, upper extremity).

Surgeries took between 269 and 587 minutes (mean 346 minutes), mean pump time was 106 minutes. During limb perfusion, three patients experienced transient hypotension and one patient went into cardiac arrest at the time of extubation. However, the majority (14/17, 82%) of patients tolerated the procedure well without significant intraoperative complications.

### Volume status

According to the anesthesia records, there was a wide range of volume given throughout the procedure (up to 13,000 cc given in an unusual case), and losses up to 2,800 cc of blood. The volumes of fluid losses from the exsanguinations of the perfused limbs were difficult to assess and were not recorded. Usually the amount of blood loss can be estimated from the volume of blood being given to the patient either systemically to maintain adequate MAP or through the perfusion circuit.

### Changes in hemodynamics

**Figure 1:**
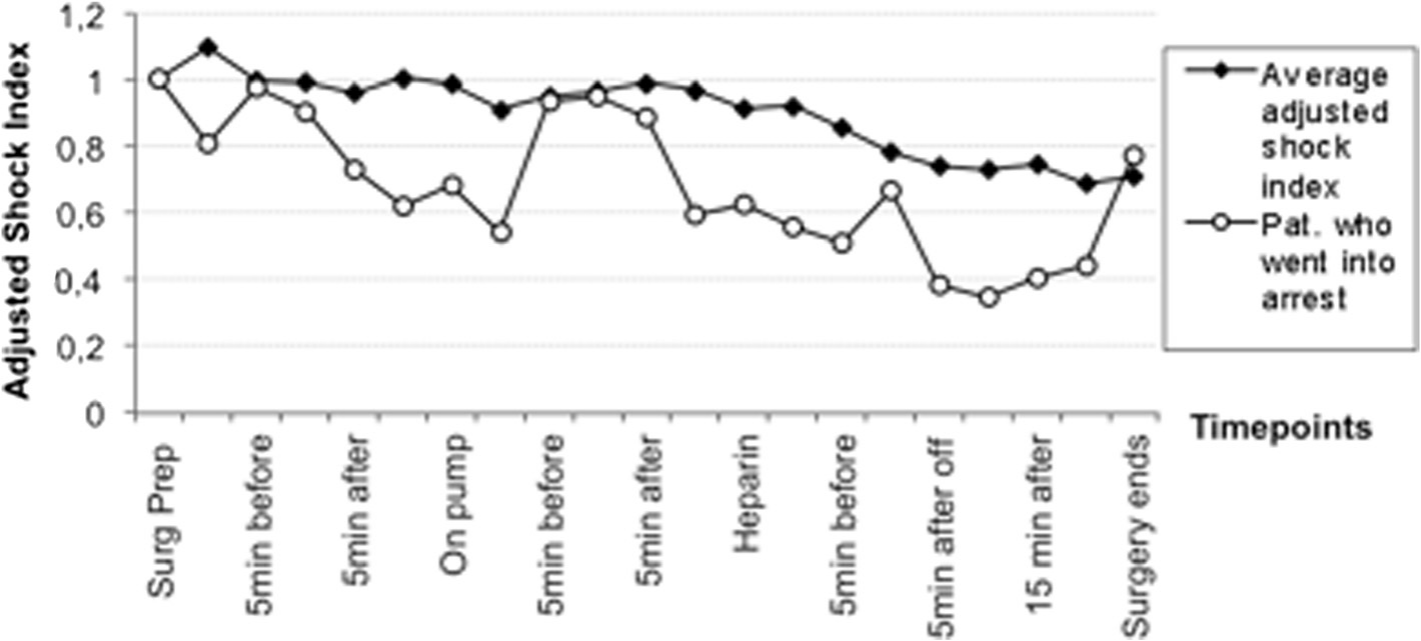
**Shown are the adjusted schock indices of 16/17 patients who tolerated the treatment well throughout all periods of the procedure.** The course of the patient who went into circulatory arrest due to poor cardiac function, fluid imbalance, masked by a paced heartrate is displayed in comparison. All patients (n=16+1*).

The adjusted MAP remained comparably stable in most stages of the procedure. Before unclamping the isolated and exsanguinated limb, pressors and colloid infusions were ready to be given in order to avoid hypotension. The adjusted shock index (HR/SBP) decreased during the cases. However, there were no significant differences to be observed (Figure 1).

The patients received between one and six units of packed red blood cells. In retrospect, both fluid balances and hematocrit changes did not appear to be suitable for statistical analysis due to incomplete data material; hematocrit values showed a trend towards slight decreases, but remained above 30%.

### Perioperative complications

One patient (ASA III) who had congestive heart failure (CHF) due to coronary artery disease and arrhythmias before, and whose HR was regulated with an automated implanted cardioverter-defibrillator went into severe hypotension at the end of his surgery and required immediate pharmacologic resuscitation and chest compressions. He responded instantly to the measures taken, and recovered without any neurological deficit. Anaphylaytic reaction to protamine was unlikely as the protamine test dose was well tolerated. His shock index as compared to other individuals was lower as showed in Figure 1. This patient also suffered from obesity, diabetes, chronic renal insufficiency and anemia.

Postoperative complications occurred in six patients and were mostly related to surgical wound infections (see Table 1). Except for one patient (no. 6), regional limb toxicity in all other patients consisted of only mild limb erythema and edema (Grade I-II) [13], without significant sensorimotor deficits. No patient developed compartment syndrome or required surgical debridement or amputation for tissue loss (Grade IV-V). With respect to oncologic outcome, at 3 months, nine of the seventeen patients (53%) were completely free of disease.

## Discussion

Cutaneous melanoma is the sixth most common cancer in the United States and an increasing health care problem worldwide [14]. Approximately half of all newly diagnosed cases of melanoma occur in the upper or lower limbs. HILP is a valuable therapeutic modality for melanoma patients with heavy disease burden confined to an isolated limb. The technical aspects of limb perfusion were first described by Creech et al. in the 1950s [5]; the procedure was subsequently modified to include hyperthermia, which enhances the cytotoxic effect of melphalan [15]. Although to date there have been no randomized controlled trials for in-transit disease of the limb which have shown prolonged survival with HILP, this procedure provides locoregional disease control in a significant percentage of patients that would otherwise require amputation [16,17].

Other treatments may include isolated limb infusion (ILI) or intralesional injection with substances such as interleukin-2 [18,19] and other agents [20]. ILI is less invasive, allows for shorter surgery and perfusion times and uses lower temperatures and might be an option for patients in otherwise limited medical and cardiac condition. Beasley (23) *et al.*[21] report an overall response rate of 61% (33% complete response, 28% partial response).

The hemodynamic changes in these procedures are important, and are known from major surgeries involving the pathophysiology of ischemia and reperfusion. The underlying cardiovascular condition with diminished cardiovascular reserves in some of the patients contributed further to the challenging anesthetic management.

In the setting of HILP, however, it is crucial for the anesthesia team need to understand that for a while two separate circulations exist. When compared to vascular or orthopedic surgery, circulation of the perfused limb requires special attention due to the ongoing perfusion. Although it is a goal to keep the perfused limb isolated from systemic circulation, volume shifts into systemic circulation can occur: melphalan can cause nausea, allergic reaction and cardiac arrest, and affect the hematopoietic system from the unavoidable leak. The influence of mediators such as cytokines that might be generated during HILP and their possible effects in systemic circulation have not been quantified. While HILP allows for delivery of higher amounts of melphalan to the disease-affected limb, regional limb toxicity is seen. In the 1980s, Wieberdink et al. described a grading scale (I-V) for toxicity associated with limb perfusion [13]. Based on larger published series, mild erythema or edema (Grade I-II) is seen in most patients [22,23]; our experience was similar. Higher grade toxicity requiring fasciotomy (for compartment syndrome) or amputation (for tissue loss) has been reported to occur in up to 1-2% of patients undergoing HILP [21,22,24].

Tumor necrosis factor alpha (TNFα) has been examined both as an additive to HILP with melphalan in sarcoma surgery [25], and also its effects on the formation of cytokines such as interleukin 6 and interleukin 8 [26], the latter being more pronounced when TNFα was added for therapeutic purposes. Christoforidis *et al.*[27] (2003) found hemodynamic responses similar to septic shock when TNFα was used during HILP procedures. TNFα has no approval for perfusion in the United States. The effects of “natural” TNFα effects may however contribute to the shock-like phenomena observed.

For the procedure of the patient who went into arrest, we suspect that his limited cardiac condition was the major cause. With the cardioverter device running at a fixed rate the limited hemodynamic response to volume shifts may have been masked. The overall worse adjusted shock index of this patient as compared to the remaining 16 patients indicates the hemodynamic instability during the case. Other patients with comparable profiles of coexisting diseases tolerated the procedure and the anesthetic well. However, in a similar situation we would advocate the use of transesophageal echocardiography to better monitor the patient’s cardiac function and fluid status. In retrospect, due to his limited condition, choosing ILI instead of HILP might have been better tolerated.

Data were retrieved in retrospect. Literature regarding the anesthetic management of this procedure is sparse. We identified milestones events in the procedure that could be found in each case. Because of the wide range of parameters, we displayed the hemodynamic data as adjusted values which, with a low total n, did not show significant results, but trends that deserve further consideration.

For HILP a team approach is crucial during which each member of the surgical team is aware of the particular aspects of each stage of the procedure. The changes in the volume status, have to be monitored invasively and require proactive and prompt reaction. We would nonetheless propose a protocol of blood tests performed before, during and after the surgery with respect to the surgical milestones to have a more standardized approach (Table 2) which also is useful for further studies.

**Table 2:** Proposed protocol for perioperative and intraoperative blood testing

	**CBC**	**Hb/Hct**	**liver function**	**ABG**	**Coagulation (PTT, INR)**	**ACT**
PREOP prepare clinic	x		x		x	
INTRAOP After incision		x		x		x
Pre-cannulation		x		(x)		x
During perfusion (q 30)		x		x		x
…		x		x		x
After washout		x		x		x
After decannulation/reestablished circulation		x		x		x
End of surgery		x		x		x
POSTOP Arrival PACU/ICU	x			x	x	(x)

Patients with preexisting cardiovascular disease should have a thorough preoperative cardiological evaluation. Continuous intraoperative assessment of cardiac function and fluid status using an echocardiography probe might be helpful. Using an indicator dilution technique might be questionable, as the algorithms for hemodynamic calculations with a PICCO catheter or a Swan-Ganz catheter use size and weight. With the clamped extremity and abnormal temperatures these might lead to wrong calculations.

It is important to be careful about blood, urine and other body fluids secretions. The washout from the extremity, the pump filling and the circuit were put into cytostatic waste cans. As a routine, we used special-safety trash cans for every secretion and urine during the first 24 hours in recovery and in the ICU.

## Conclusion

HILP is a treatment option in an otherwise desperate situation for patients with invasively growing melanoma in an extremity. The procedure requires thorough patient preparation, a dedicated, experienced surgical, perfusionist, nursing and anesthesia team, a perioperative setting that allows for intensive monitoring and care throughout the entire procedure and the postoperative period. High risk patients with significant cardiac compromise should probably not be candidates for HILP. We suggest a standard treatment scheme of monitoring, blood tests and record keeping especially with respect to hemodynamic and volume changes.

## Competing interests

No author had any competing interests.

## Authors’ contributions

During their collaboration at UCSF, HR and SL had the idea to analyse the cases. They wrote introduction, results and discussion and edited the entire manuscript. SS, KK and CA collected and analysed the data material. WT and SL wrote the section of the surgical methods and provided clinical data about the clinical results. PC and DS provided methdological information regarding vascular access and extracorporeal circulation. MKS and DM contributed to the discussion section regarding therapeutic options and outcomes. All authors read and approved the final manuscript.

## Pre-publication history

The pre-publication history for this paper can be accessed here:

http://www.biomedcentral.com/1471-2253/13/15/prepub
